# Cognitive Agents in Urban Mobility: Integrating LLM Reasoning into Multi-Agent Simulations

**DOI:** 10.3390/s25185688

**Published:** 2025-09-12

**Authors:** Christian Calderón, Pasqual Martí, Jaume Jordán, Javier Palanca, Vicente Julian

**Affiliations:** 1 Valencian Research Institute for Artificial Intelligence, Universitat Politècnica de València (UPV), Camino de Vera s/n, 46022 Valencia, Spain; ccalderon@upv.es (C.C.); pasmargi@vrain.upv.es (P.M.); jjordan@dsic.upv.es (J.J.); jpalanca@dsic.upv.es (J.P.); 2 Valencian Graduate School and Research Network of Artificial Intelligence (VALGRAI), 46022 Valencia, Spain

**Keywords:** urban mobility simulation, large language models, agent-based modeling, adaptive behavior, mobility disruptions

## Abstract

Urban mobility systems face escalating challenges associated with sustainability, equity, and resilience, further compounded by environmental pressures. Traditional agent-based models (ABMs) often fail to capture cognitively rich, adaptive behaviors, limiting their ability to simulate realistic user responses to disruptions. In this work, we propose a cognitive agent architecture based on Large Language Models (LLMs), featuring multi-horizon memory-driven planning, reflection, and adaptation. Integrated into the SimFleet agent-based simulator with realistic sociodemographic profiles, the agents dynamically generate, adjust, and reflect upon travel plans across a 20-day simulation involving over 320 individuals. Experimental results reveal emergent adaptation patterns under both stable and disrupted transport conditions, and an ablation study under severe service disruption quantifies the contributions of short-term and long-term memory modules to memory-driven reasoning, demonstrating the potential of LLM-driven agents to enhance the realism, flexibility, and interpretability of urban mobility simulations.

## 1. Introduction

The rapid expansion and increasing complexity of urban environments have transformed mobility from a logistical challenge into a multidimensional issue encompassing sustainability, equity, and resilience. Cities are now dense ecosystems, characterized by dynamic population flows, multimodal transport systems, and frequent disruptions caused by climate events, policy changes, and social dynamics. According to the World Social Report 2024 [1], achieving equitable and sustainable urban development requires mobility systems that are not only efficient but also adaptable to socioeconomic diversity and exogenous shocks. Recent studies confirm that mobility inequality and accessibility gaps remain persistent challenges in urban systems, often exacerbated by environmental and infrastructural constraints [2,3].

In parallel, environmental concerns are increasingly prominent. While some major cities such as Beijing and Shanghai have achieved significant improvements in air quality, many emerging metropolitan areas across Latin America, Africa, and Southeast Asia continue to suffer from high pollution levels linked to modal inefficiencies and poor transport integration. Traffic congestion, in particular, remains a dominant externality of urban mobility systems. Faheem et al. [2] project that congestion-related economic losses will exceed USD 96 billion annually in the United States by 2030.

Xia et al. [3] demonstrate that congestion peaks correspond with localized surges in NO_2_ and CO levels, creating hazardous urban microclimates that intensify public health risks. To address these issues, simulation tools have become central to transportation research and urban planning. Agent-based modeling (ABM) stands out due to its capacity to represent heterogeneous behaviors and interactions within complex adaptive systems [4,5]. Despite its promise, however, ABM suffers from several structural limitations. A recent review [4] highlights three recurring gaps: (i) reliance on static, rule-based behavior models; (ii) insufficient incorporation of adaptive learning; and (iii) lack of emphasis on equity and resilience metrics. Even recent advances in hybrid approaches—such as those incorporating imitation learning [6] or supervised models like GBDT [7]—fail to overcome core limitations related to cognitive flexibility, interpretability, and generalization to disruptive contexts.

Emerging Large Language Models (LLMs) offer an alternative paradigm for modeling human decision-making, enabling simulation agents to reason, reflect, and adapt through natural language interaction. Recent work demonstrates that LLMs can generate realistic travel plans [8], predict multimodal travel behaviors [9], infer semantic purpose from trajectory data [10], and explain behavioral adaptation under disruption [11]. Architectures such as ExpeL [12] introduce experiential learning mechanisms where agents retrieve episodic memory and synthesize semantic patterns from prior interactions, supporting adaptation without parameter tuning. Gong et al. [13] further illustrate how LLMs can generate trip patterns by combining intentions, preferences, and contextual constraints into cognitively plausible behaviors. This progression supports a new class of urban mobility agents capable of simulating not only action but cognition. Agents can now plan weekly routines, reflect on daily choices, adjust decisions in response to exogenous events, and store generalized knowledge for future tasks. These advances align with recent developments in interpretable reasoning [14], multimodal planning [15], and simulation frameworks that incorporate user prompting [16]. In particular, systems such as ChatSUMO [16] and TransCompressor [14] highlight the feasibility of integrating urban simulations with conversational interfaces, enabling flexible, explainable, and responsive agent behavior.

Beyond the cognitive architecture itself, modeling mobility must also reflect sociodemographic heterogeneity. Numerous studies underscore the relevance of attributes such as age, gender, occupation, and educational background in shaping modal preferences and travel behavior [17,18,19]. Li et al. [9] apply BERT-based embeddings to identify over 30 distinct multimodal travel patterns, with strong links to demographic clusters. Ma et al. [20] propose a framework of AI mobility agents embedded in evolving networks, highlighting the dynamic feedback between individual decisions and system-wide transport performance. Our work adopts this perspective by incorporating a set of diversified agent profiles to simulate heterogeneous and adaptive mobility responses (see Section 5 for details). The implementation of our framework is grounded in SimFleet, a validated agent-based mobility simulator also used in [21] for bus ridership prediction under machine learning scenarios. In contrast with that study, which focuses on predictive modeling, our framework implements a cognitively enriched decision cycle. By cognitive enrichment of the decision cycle, we mean that agents move beyond fixed rules by combining identity-driven weekly intentions with daily reflection informed by episodic feedback and environmental context. In this way, the four profiles exhibit heterogeneous yet interpretable adaptations under identical conditions (see Section 4 and Section 5). This setup enables the exploration of complex scenarios, such as service disruptions, modal restrictions, and policy interventions, under conditions of agent-level reasoning and learning.

We adopt Valencia, Spain, as a reference case study because it reflects the mobility challenges of a medium-sized European city. The city combines dense central districts with peripheral neighborhoods, a multimodal transport system centered on buses, and growing reliance on on-demand mobility such as taxis and private hire vehicles. Local reports highlight recurrent congestion in major corridors and capacity constraints on some lines (e.g., Line 10), which undermine reliability and equitable access. These features make Valencia a representative testbed for analyzing adaptive behaviors under disruption, with insights transferable to other urban areas of similar scale.

This study does not rely on empirical mobility datasets from Valencia; instead, it uses stylized behavioral archetypes—students, administrative workers, elderly residents, and factory workers—derived from survey-based mobility research. These archetypes capture empirically observed tendencies in modal choice, cost and time sensitivity, and comfort requirements, allowing controlled testing of the cognitive framework while remaining aligned with real-world behavioral evidence.

Beyond its methodological novelty, the relevance of this study is twofold. Scientifically, it advances urban mobility simulation by embedding cognitively inspired mechanisms—short- and long-term memory, reflective reasoning, and semantic preferences—into agent-based models, overcoming the rigidity of rule-based approaches. Practically, it addresses challenges faced by transport planners and policymakers, including anticipating how diverse user groups adapt to disruptions, service changes, or policy interventions. By enabling agents to form routines, reflect on disruptions, and adapt strategies over time, the framework offers a transparent and extensible tool for evaluating resilience, equity, and behavioral plausibility in mobility systems.

Specifically, this paper makes the following original contributions:

- We introduce a cognitive agent architecture for urban mobility that leverages LLMs with multi-horizon planning (long-term weekly plans based on identity and short-term daily adaptations using episodic feedback and environmental context), episodic memory consolidation, and semantic abstraction.
- We develop a profile-based simulation environment incorporating realistic sociodemographic attributes, enabling heterogeneous, context-sensitive agent behaviors across multimodal urban scenarios.
- We integrate the cognitive framework into SimFleet, extending the platform to support cognitively enriched agents capable of reflective adaptation to environmental disruptions and personal preference shifts.
- We validate the architecture via a 20-day simulation of 320 agents in baseline and disruption scenarios, and perform an ablation study to quantify the impact of short-term and long-term memory modules.

The remainder of the paper is structured as follows: Section 2 reviews the related work on agent-based modeling, cognitive architectures, and LLM-driven simulations. Section 3 presents SimFleet, the underlying urban mobility simulator. Section 4 details the proposed cognitive architecture and its integration into SimFleet. Section 5 describes the experimental setup and simulation design. Section 6 presents the experimental results. Section 7 discusses broader implications, limitations, and interpretability challenges. Finally, Section 8 concludes the paper and outlines directions for future research.

## 2. Related Work

Advances in urban mobility simulation increasingly draw from developments in agent-based modeling, machine learning, and cognitive architectures. This section analyzes related work relevant to the development of adaptive and cognitively enriched mobility simulation agents.

### 2.1. Limitations of Traditional ABM and ML Approaches

Agent-based models (ABMs) have been instrumental in simulating urban mobility systems due to their ability to represent heterogeneous agents, spatial environments, and interaction dynamics. However, several limitations persist in conventional ABM frameworks. As Divasson-Jutgla et al. [4] outline in their systematic review, most transportation-focused ABMs rely on static behavioral rules, lack contextual adaptation, and do not incorporate memory or reflection mechanisms. In this regard, Martí et al. [22,23] introduced generators for fleets, charging stations, and mobility data to enhance the realism and configurability of simulation environments. While such contributions expand the scope of scenario construction and input variability, they remain input-oriented and do not confer adaptive or reflective capacities upon agents themselves. The rule-based nature of these agents restricts their ability to generalize or respond to unexpected events, such as infrastructure failures, modal shifts, or policy interventions. Moreover, hybrid approaches that combine ABMs with machine learning have made important advances but remain limited in cognitive capabilities. For example, Wang et al. [6] present PateGAIL, a model that employs generative adversarial imitation learning to generate synthetic mobility trajectories while preserving privacy. Although effective in trajectory synthesis, the approach is devoid of semantic planning or reflective capabilities.

Similarly, Zhao et al. [7] propose a GBDT-based classifier for trip purpose prediction using survey and POI data. While achieving solid prediction accuracy, their model depends on supervised learning and predefined labels, lacking adaptability to new environments or behaviors. Martí et al. [21] integrate ML methods such as Random Forest and SVR with the SimFleet simulation environment to predict bus ridership. Their study validates SimFleet’s robustness for multimodal simulation but operates within a predictive modeling paradigm that lacks reasoning or memory. In all these cases, agents are treated as data-driven black boxes or rule-based executors, not as cognitive entities capable of reflecting on past actions or modifying their strategies in response to experience.

These limitations justify the need for a new class of simulation agents that combine learning, reasoning, memory, and adaptability within urban environments. To illustrate these structural gaps more explicitly, Table 1 contrasts the functional scope of traditional rule-based ABMs, hybrid ABMs incorporating machine learning, and our proposed LLM-based cognitive agent model. While hybrid methods may increase predictive flexibility, they do not address core cognitive limitations such as memory, multi-horizon planning, or reflective adaptation. These features are structurally incompatible with rule-based logic systems and cannot be retrofitted into ML-enhanced agents without altering their fundamental architecture.

### 2.2. Cognitive Architectures with LLMs

Recent developments in LLMs have sparked a new generation of cognitive agents capable of integrating memory, planning, and contextual adaptation. Unlike traditional agent-based simulations, these architectures simulate not only action sequences but also the internal reasoning processes underlying decision-making. One representative example is ExpeL [12], which introduces a dual mechanism of experiential learning: episodic recall of successful behaviors and semantic abstraction of strategic insights.

The agent does not rely on parameter updates but instead reuses natural language memories to improve across tasks. The importance of memory modeling is emphasized by Hatalis et al. [24], who identify the cognitive necessity of separating episodic, semantic, and procedural memory to ensure coherence and reduce hallucinations. They argue that LLM agents using vector-based memory structures must be equipped with semantic indexing and retrieval to sustain contextual continuity. Hou et al. [25] extend this paradigm with a time-aware recall model that uses memory decay functions, frequency-based reinforcement, and context-aware prompts to emulate human-like consolidation. Beyond task-specific applications, Hou et al. [25] propose a memory-aware conversational agent that integrates episodic storage and frequency-based recall to enhance contextual relevance. While not designed for urban mobility, their framework supports dynamic prompting and agent continuity, reinforcing the importance of memory modeling in cognitive agents. Similarly, Hatalis et al. [24] present a theoretical taxonomy of long-term memory for LLM agents, arguing for the separation of episodic, semantic, and procedural knowledge to mitigate hallucinations and preserve decision consistency. These contributions, although conceptual, inform the structuring of memory systems within cognitively enriched agent architectures.

In terms of symbolic generalization, Gong et al. [13] present a model where travel preferences are embedded semantically and used to guide trip generation in LLM agents. Similarly, Zhang et al. [8] propose MobGLM, a transformer-based LLM trained on multimodal travel patterns, achieving superior realism and generalizability across user profiles. Zhong et al. [11] focus on robust forecasting under disruption, transforming time series into semantically enriched prompts, demonstrating that LLMs outperform traditional predictors even in irregular settings.

Paul [26] contributes NeoPlanner, a hybrid planning framework that combines reinforcement learning with LLM-guided symbolic reasoning. The system constructs POMDP graphs from interactions and stores entity-relationship triplets as long-term memory for task transfer. This echoes the consolidation flow in our model, where episodic traces are abstracted into reusable knowledge patterns. Finally, Zhang et al. [27] introduce MindMemory, an LLM agent architecture with structured long-term memory separated into episodic, semantic, and abstract modules. Their model outperforms GPT-4 variants in longitudinal memory tasks and highlights the role of personality persistence and memory-driven decision consistency.

Collectively, these architectures demonstrate the feasibility and value of embedding LLMs within structured memory systems for urban simulation agents. Our work builds upon this foundation by extending cognitive planning into a multi-agent urban environment with explicit sociodemographic variability and iterative reflection, designed to handle daily disruptions and emergent behavior over time.

### 2.3. Sociodemographic Profiles and Multimodal Travel Patterns

Understanding urban mobility requires attention not only to spatial and modal factors, but also to the sociodemographic characteristics that shape individual travel preferences and constraints. A growing body of literature highlights the need to integrate demographic segmentation into simulation models to capture the diversity of behavioral patterns. Huang et al. [17] emphasize gender-based differences in transport decisions, noting that safety, accessibility, and reliability are prioritized differently by men and women. Jafarzadehfadaki et al. [18] employ a cluster-based analysis to show how age, income, and employment status jointly influence mode choice and trip frequency. Yang et al. [19] analyze public transport usage across demographic groups and find substantial variation in access, frequency, and satisfaction levels. Their results support the use of differentiated agent profiles to simulate realistic urban mobility dynamics.

In a more formalized approach, Li et al. [9] leverage BERT-based embeddings to classify over 30 distinct multimodal travel patterns from trajectory data. These patterns align strongly with demographic clusters and suggest that semantic representation of agent behavior enhances model generalization. Ma et al. [20] extend this view by proposing mobility agents embedded in evolving AI networks, where decision feedback loops are conditioned on demographic and temporal attributes. Their framework points to the value of context-aware adaptation, where agents not only choose based on static preferences but also learn from accumulated experience.

Our own framework adopts these principles by designing four sociodemographic agent profiles with varying preferences, constraints, and adaptability to disruptive scenarios. This structure facilitates experimentation on how diverse populations respond to mode changes and environmental perturbations, providing a richer and more inclusive simulation of urban mobility systems.

### 2.4. Open Challenges and Research Gaps

While recent advances in LLM-based cognitive agents represent a significant leap forward, several research gaps remain unaddressed. First, most existing architectures are designed for isolated tasks or synthetic environments, lacking integration with realistic urban simulation platforms. Few studies explore how cognitive agents behave in multimodal transport systems or respond to policy changes and external disruptions. Moreover, although memory structuring is increasingly discussed [24,25,27], the practical implementation of dynamic episodic-to-semantic consolidation in urban contexts remains underexplored.

Another limitation concerns the underrepresentation of diverse populations in agent-based LLM frameworks. While recent works include basic demographic variations [9,18], there is a lack of systematic modeling of marginalized or mobility-constrained users. Additionally, most studies focus on prediction or replication of behavior, rather than on agent-level learning, reflection, or ethical adaptability over time. Lastly, although some frameworks integrate LLMs with planning or reinforcement learning modules [26], the real-time interaction between planning, memory, and environment adaptation is still nascent. Our work addresses these limitations by combining memory-aware LLM agents, rich sociodemographic profiling, and a validated simulation platform (SimFleet) to explore reflective planning in a realistic, multimodal, and dynamically disrupted urban setting.

### 2.5. Comparative Positioning of LLM-Based Mobility Agents

To assess the novelty and functional scope of our proposed cognitive framework, we present a comparative analysis of 13 relevant architectures, spanning agent-based models (ABMs), machine learning predictors, imitation-based generators, and recent LLM-driven cognitive systems. The comparison is structured into two analytical axes: (i) cognitive capabilities and (ii) application context. Table 2 and Table 3 summarize this analysis. We classify each capability or feature as ‘Yes’, ‘No’, or ‘Partial’ based on the presence, absence, or limited implementation of that property, as described in the corresponding original works. For instance, Partial adaptation denotes implicit adjustments (e.g., few-shot prompting) without structured memory or decision feedback, while ‘Yes’ indicates mechanisms such as memory-driven re-planning or explicit behavioral revision.

Cognitive capabilities. As shown in Table 2, only a subset of reviewed architectures implement structured memory systems (e.g., ExpeL, MindMemory, NeoPlanner), and even fewer support multi-horizon planning or dynamic adaptation. Most existing ABM or ML-based systems (e.g., Divasson [4], Zhao [7]) lack reflexive behavior, rely on static rules or supervised predictors, and do not simulate human-like learning processes. In contrast, our agents integrate episodic memory, semantic abstraction, and daily reflective cycles that enable long-term behavioral evolution. Furthermore, our architecture includes explicit sociodemographic profiles, allowing simulation of heterogeneous behavior across modal preferences—an aspect underrepresented in LLM-agent research.

Application context. Table 3 shows that most cognitive architectures were developed for synthetic tasks (e.g., NeoPlanner [26]), dialogue agents (e.g., MindMemory [27]), or theoretical proposals (e.g., Hatalis [24]). Among mobility-oriented models, Mobility-LLM [13], MobGLM [8], and HMP-LLM [11] focus on preference inference or forecasting but do not simulate agents or disruptions.

TransCompressor [14], while not a cognitive agent model, uses LLMs to reconstruct multimodal transport data via structured prompting. It contributes to transport system intelligence but does not engage in planning or behavioral simulation.

Positioning. Our framework combines LLM-based memory structuring, multi-horizon planning, reflective adaptation, and sociodemographic agent profiling in a validated agent-based mobility simulator. It enables cognitively plausible decision-making under urban constraints, offering a novel pathway for simulating adaptive, explainable, and heterogeneous behavior in complex transport environments.

## 3. SimFleet: Urban Mobility Simulator

SimFleet is an agent-based simulator built upon SPADE, purposefully developed to model, evaluate, and optimize urban mobility services (SPADE 4.1.2 version (Smart Python Agent Development Environment) is an agent-oriented programming library that supports FIPA-compliant communication protocols and asynchronous message passing in distributed systems). It permits the integration of various transportation modalities—including taxis, buses, pedestrians, and electric vehicles—within a cohesive simulation environment. Initially conceptualized as a multi-agent fleet simulator [28], SimFleet has since undergone substantial architectural enhancements to meet modern demands for scalability, extensibility, and behavioral flexibility.

### 3.1. Motivation and Architectural Evolution

The original version of SimFleet was designed to coordinate urban taxi fleets and relied on a centralized agent architecture, with route planning handled externally and behavioral logic embedded within agent classes. While this approach demonstrated agent-based interaction, it was limited to a single transport mode, hindered extensibility, and did not accommodate emerging urban mobility paradigms such as electric vehicles or micromobility. To overcome these limitations, SimFleet has been fundamentally redesigned as a modular, multimodal simulation platform. Key architectural innovations include decentralized route planning (integrated via the MovableMixin), mixin-based composition of capabilities, and modular, strategy-driven behavioral logic. These features allow for greater flexibility, scalability, and realism in modeling heterogeneous urban mobility scenarios. Certain concepts underpinning this redesign—such as the use of mixins and transport-specific strategy patterns—were first explored experimentally in previous work [29]. However, that study focused on a specific application scenario and did not formalize a general-purpose architecture.

The redesigned SimFleet architecture introduces a modular class hierarchy structured around role specialization and functional composition. Rather than relying solely on rigid inheritance chains, the system employs mixin-based design patterns to decouple capabilities from core agent types. This enables the flexible construction of heterogeneous agents by dynamically attaching functional traits relevant to the agent’s role in the system, without requiring deep class nesting or code duplication.

The overall design follows object-oriented composition principles, allowing agents to be defined by their roles and capabilities rather than by strict taxonomic categories. For example, both vehicles and autonomous pedestrians may share navigational logic through a common mobility module, while energy-based behaviors are composed only when applicable (e.g., for electric vehicles). This layered design facilitates extensibility and supports realistic simulation of multimodal environments.

### 3.2. Functional Composition via Mixins

A central innovation in the current architecture is the use of mixins—lightweight, reusable class fragments that encapsulate specific functionalities. Mixins are applied to base agent classes to endow them with additional behaviors in a composable manner.

Two primary mixins currently define key capabilities in SimFleet:

- MovableMixin, which provides autonomous navigation, spatial localization, and route-following logic;
- ChargeableMixin, which adds support for energy consumption tracking, battery capacity, and interaction with charging stations.

**Figure 1:**
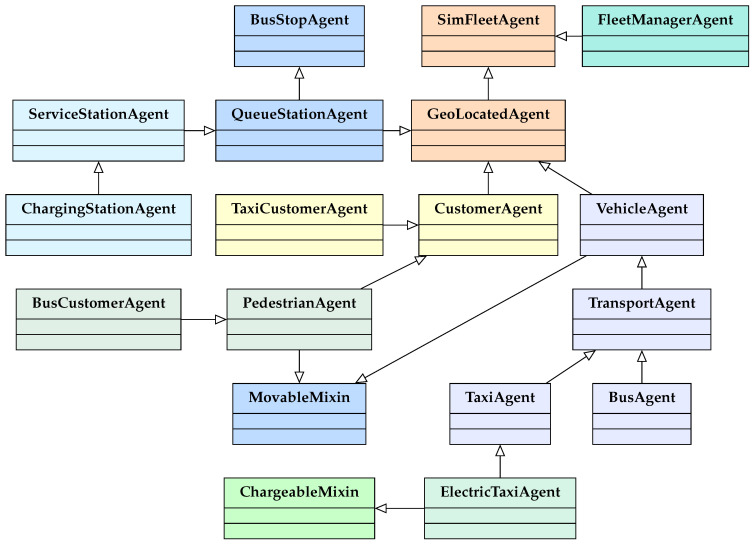
SimFleet class architecture diagram. Colours indicate agent categories: core/transport agents (orange tones), infrastructure and station agents (blue tones), user/customer agents (yellow), mixins (green), hybrid roles such as vehicle/transport (blue–orange family), pedestrian/customer hybrid (light green), and electric variants (green–teal). The colours are used solely to aid readability; all node labels define the functional role in the hierarchy.

These mixins can be combined with any agent that requires such capabilities. For instance, the ElectricTaxiAgent class inherits from TaxiAgent and composes both MovableMixin and ChargeableMixin, resulting in a fully mobile, energy-aware transport agent. This approach avoids redundant implementations across agent types and enables the simulation of diverse roles with shared functionalities. For example, pedestrian agents can also be made mobile by composing the MovableMixin, even if they are not vehicles. This design pattern significantly improves the flexibility of agent creation and maintains a clean, modular codebase. These compositional relationships are visually represented in the class hierarchy diagram (Figure 1), where the structural layering of core agents, mixins, and specializations can be observed.

### 3.3. Agent Roles and Hierarchies

SimFleet defines its agents through a structured class hierarchy based on functional roles and transport modality. At the core lies the abstract SimFleetAgent, which serves as the base class for all agents within the system. This base is extended in two primary directions: spatially embedded agents and coordination-oriented agents. The GeoLocatedAgent class introduces spatial awareness and serves as the foundation for agents that operate within the simulated geography. This includes the following:

- CustomerAgent, with further specializations such as TaxiCustomerAgent,
  BusCustomerAgent, and PedestrianAgent;
- VehicleAgent, which generalizes transport-capable agents and is extended into specialized types like TransportAgent, TaxiAgent, BusAgent, and ElectricTaxiAgent.

Meanwhile, the FleetManagerAgent derives directly from SimFleetAgent and is responsible for orchestrating agent interactions and managing service coordination policies. This hierarchy promotes reuse and separation of concerns. For instance, a pedestrian agent and a bus customer may share the same spatial capabilities but differ in mobility traits and interaction logic. The design supports complex multimodal interactions by allowing each agent to inherit only the behaviors and capabilities required for its role.

The hierarchical relationships among these agents are visually represented in the class diagram (Figure 1), which highlights the inheritance structure and compositional logic across agent types. This role-based structure has also been successfully applied in real-world simulation scenarios. For instance, in Martí et al. [21], the hierarchy enabled the modeling of public transport services using BusCustomerAgent, BusAgent, and BusStopAgent roles. This configuration allowed demand-driven evaluations of passenger flows and coordination strategies, demonstrating the architecture’s applicability to practical urban mobility studies.

### 3.4. Infrastructure and Environment Integration

The simulation environment in SimFleet is populated not only by mobile agents but also by fixed infrastructure components that support transport operations and service logistics. These are implemented through specialized station agents, which interact with vehicles and customers to model realistic service points such as charging stations, depots, or bus stops.

The infrastructure design is grounded in two core agent classes:

- QueueStationAgent, which provides queuing logic for boarding and waiting areas;
- ServiceStationAgent, which generalizes infrastructure entities such as maintenance depots or utility access points.

These core classes are extended into application-specific roles, for example, as follows:

- BusStopAgent extends QueueStationAgent to support customer boarding logic and bus arrival scheduling;
- ChargingStationAgent extends ServiceStationAgent and interacts with
  ElectricTaxiAgent to manage energy replenishment cycles.

All infrastructure agents are spatially positioned within the simulated map and may incorporate parameters such as maximum capacity, service times, or queue discipline. Real-world locations can be derived from OpenStreetMap (OSM) data, allowing simulations to reflect actual urban topologies. Agents interact with infrastructure components using geospatial proximity and event-driven triggers, enabling scenarios such as dynamic congestion, access prioritization, or timed service windows. This integration between agents and environment enables the simulation of rich urban scenarios. For instance, bus stops can be configured to handle peak-hour passenger surges, and charging stations can become operational bottlenecks during high-demand periods. These interactions are especially important when evaluating infrastructure planning, load balancing, or energy distribution in smart mobility systems.

### 3.5. Behavioral Modularity via Strategy Pattern

To promote flexibility and reduce structural coupling, SimFleet adopts the Strategy design pattern to modularize agent behaviors. Instead of embedding decision-making logic directly into agent classes, behavioral strategies are defined as separate modules that can be assigned or switched at runtime. Each agent type supports one or more strategy interfaces appropriate to its role, for example, as follows:

- TaxiAgent and BusAgent may implement dispatch or routing strategies;
- FleetManagerAgent can employ coordination strategies for assigning tasks or balancing service load;
- PedestrianAgent may follow navigation strategies that incorporate shortest-path, attraction-based, or stochastic behaviors.

This approach enables the implementation and testing of diverse behavioral models without altering the core agent architecture. Strategies can be changed during simulation time to evaluate how different logic structures affect system-level outcomes such as efficiency, travel time, or resource usage [29]. The modularity provided by this pattern facilitates controlled experimentation and comparative analysis. For instance, in [21], different dispatching strategies were evaluated in terms of service efficiency and demand coverage by altering only the behavioral modules, while maintaining identical agent and environment configurations. This decoupling makes SimFleet particularly suitable for testing and comparing coordination mechanisms in urban mobility scenarios. Technical implementation details, including configuration structure, routing logic, and execution lifecycle, are provided in Appendix A to support reproducibility and transparency.

## 4. Cognitive Architecture for Mobility Simulation

This section introduces our cognitively enriched mobility simulation framework. Building upon the modular and multimodal capabilities of SimFleet, we embed reflective agents capable of long-horizon planning and short-term adaptation using LLMs, episodic memory, and semantic abstraction. The proposed architecture integrates individual preferences, environmental context, and experience-based reasoning to simulate dynamic, human-like transport behaviors.

### 4.1. Conceptual Model of the Cognitive Agent

**Figure 2:**
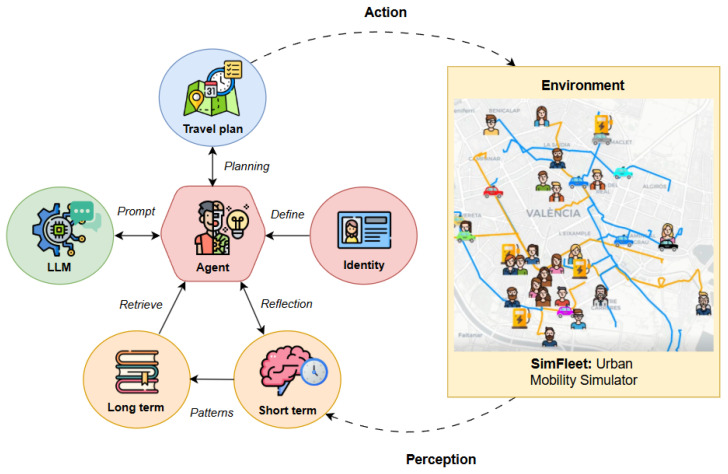
Conceptual architecture and feedback loop of the cognitive agent in SimFleet.

Figure 2 presents a high-level overview of the cognitive agent’s architecture and its interaction with the simulation environment. The agent operates through a cyclical loop of planning, execution, observation, and reflection, enabling it to adapt dynamically to environmental changes and behavioral feedback. At the core of the agent is its identity, which defines intrinsic characteristics such as sociodemographic attributes, transport preferences, and activity schedules.

This identity shapes how the agent interprets its environment and influences its initial planning strategies.

The agent maintains two memory systems: short-term memory, which holds recent experiences from daily simulations, and long-term memory, which captures general behavioral patterns formed over time. These memory structures are key to enabling reflection and adaptation, as advocated in recent frameworks for experiential learning in LLM-based agents [12,27]. Each simulation cycle begins with the agent submitting a planned journey to the environment, represented as a travel plan. The agent observes the outcomes of this plan after execution in the SimFleet environment, including journey duration, delays, or unexpected events. The agent then engages in a reflection process, drawing insights from the observed outcomes and updating its short-term memory accordingly. Over time, consistent patterns in behavior and response are abstracted and stored in the long-term memory, enriching the agent’s capacity for future planning. This closed-loop interaction embodies a cognitively inspired decision-making framework, in which planning is not static but continuously shaped by experience and context. The next subsection provides a formal definition of this architecture and its multi-horizon planning process.

### 4.2. Cognitive Decision Loop with Multi-Horizon Memory-Driven Planning

To support adaptive and context-aware mobility behavior, we propose a cognitively inspired planning loop structured around two complementary horizons: a long-horizon weekly plan and a short-horizon daily adaptation. This dual-layered design mirrors real-world decision processes, where individuals formulate general plans and revise them daily based on experiences and environmental dynamics.

Agent State Representation. At each time step *t*, an agent *a* is represented as:

$$a_{t}=\left(I,M_{t}^{s},M_{t}^{l},p_{t},E_{t}\right)$$

- *I*: Agent identity, including sociodemographic information (e.g., age, income) and modal preferences (e.g., comfort sensitivity).
- $M_{t}^{s}$: Short-term memory, which stores episodic data from previous days, including outcomes and reflections. This memory is reset at the end of each planning cycle.
- $M_{t}^{l}$: Long-term memory, consisting of abstracted behavioral patterns and generalized strategies formed over time.
- $p_{t}$: Travel plan for day *t*, derived from the initial weekly plan or revised through daily adaptation.
- $E_{t}$: Environmental context on day *t*, including weather, infrastructure disruptions, or special events.

Long-Horizon Weekly Planning. At the beginning of each cycle (e.g., a 5-day work week), the agent generates a high-level plan:

$$\mathrm{Plan}={\mathrm{LLM}}_{\mathrm{weekly}}\left(I,M_{0}^{l}\right),\;\mathrm{where}\;\mathrm{Plan}=\left\{p_{1}^{\mathrm{init}},...,p_{N}^{\mathrm{init}}\right\}$$

Each $p_{t}^{\mathrm{init}}$ defines the intended departure time, transport mode, and destination for day *t*. The weekly plan is generated using identity and long-term memory only, reflecting intentions formulated under general assumptions.

Daily Reflection and Re-Planning. On each day *t*, the agent executes the prior day’s plan:

$$s_{t-1}=\mathrm{SimFleet}\left(p_{t-1},E_{t-1}\right)$$

The output $s_{t-1}$ includes trip-level indicators such as travel duration, delays, costs, and satisfaction scores. The agent then performs a reflective evaluation:

$$r_{t-1}={\mathrm{LLM}}_{\mathrm{reflect}}\left(p_{t-1},s_{t-1}\right)$$

This outcome is appended to the short-term memory:

$$M_{t}^{s}=M_{t-1}^{s}\cup \left\{\left(p_{t-1},s_{t-1},r_{t-1}\right)\right\}$$

The agent then adapts the current day’s plan using recent experiences, the original plan component $p_{t}^{\mathrm{init}}\in \mathrm{Plan}$, and the current environment:

$$p_{t}={\mathrm{LLM}}_{\mathrm{daily}}\left(I,M_{t}^{s},p_{t}^{\mathrm{init}},E_{t}\right)$$

The updated plan is executed in the simulation:

$$s_{t}=\mathrm{SimFleet}\left(p_{t},E_{t}\right)$$

Pattern Abstraction and Memory Consolidation. At the end of the planning cycle (i.e., after *T* days), the accumulated short-term memory is summarized and abstracted into generalized patterns:

$${\mathrm{Patterns}}_{T}={\mathrm{LLM}}_{\mathrm{abstract}}\left(M_{1:T}^{s}\right),\;M_{T+1}^{l}=M_{T}^{l}\cup {\mathrm{Patterns}}_{T}$$

Here, *T* denotes the total number of days in the cycle (e.g., T=5 for a working week). The short-term memory is then cleared, preparing the agent for the next iteration. This multi-horizon architecture supports robust behavioral realism: weekly plans provide temporal coherence and foresight, while daily reflection allows for adaptive correction. Through structured memory management and LLM-guided abstraction, agents develop both habitual patterns and responsiveness to change—key traits of human mobility behavior. To complement the formal description, Algorithm 1 provides a pseudocode summary of the cognitive decision loop. It shows how agents generate weekly plans, adapt daily strategies through reflection, and consolidate experiences into long-term memory.
**Algorithm 1:** Cognitive Agent Simulation Loop with LLM-Driven Planning.
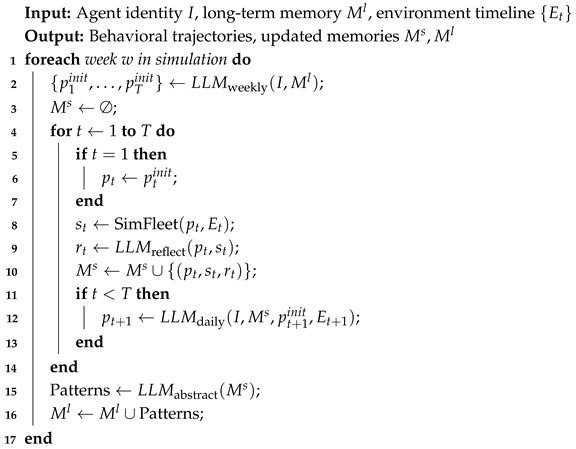


### 4.3. Structured Memory Representation and Semantic Continuity

While the framework described above distinguishes between short-term and long-term memory, these structures can be interpreted through a more fine-grained cognitive taxonomy. Short-term memory fulfils the role of episodic memory, temporarily storing recent travel experiences and reflective evaluations. Long-term memory serves as a form of semantic memory, where abstracted behavioral patterns are consolidated over successive planning cycles. A third component, procedural memory, is only partially instantiated in the present framework: repeated merging of common patterns across weeks provides a rudimentary form of procedural knowledge, whereas a more explicit modeling of habitual rules and routines is considered future work (e.g., combining LLM-generated patterns with machine learning models to stabilize procedural strategies). This tripartite view of memory supports coherence in planning and reduces the risk of spurious or inconsistent behavior, aligning with established principles of cognitive architectures.

**Figure 3:**
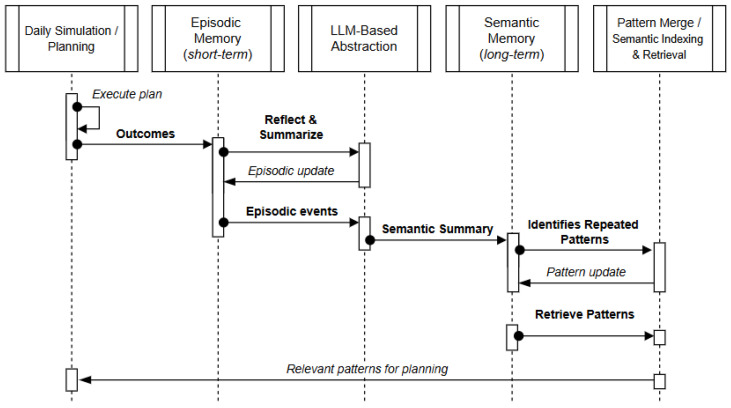
Structured memory management in cognitive agents. This UML sequence diagram complements Algorithm 1: daily outcomes are stored in episodic memory, abstracted by the LLM into semantic patterns, periodically consolidated, and progressively contribute to the emergence of procedural tendencies updated within semantic memory.

To maintain contextual continuity across cycles, memory consolidation does not simply log all past experiences. Instead, accumulated episodes are periodically summarized through LLM-guided abstraction (e.g., weekly) and selectively merged at longer horizons (e.g., monthly). This process acts as a form of semantic indexing: redundant information is compressed, salient regularities are retained, and irrelevant details are discarded. As a result, retrieval of relevant patterns for both daily adaptation and weekly planning remains tractable and contextually grounded, ensuring that agent reasoning is guided by meaningful experience rather than unfiltered historical noise. Figure 3 highlights how this layered organization operationalizes the interaction between episodic and semantic layers, showing how semantic indexing and LLM-guided abstraction maintain coherence across planning horizons.

### 4.4. Robustness and Fallback Mechanisms

To ensure execution continuity and architectural robustness, especially under real-time constraints, our framework implements two structured fallback mechanisms embedded within the cognitive decision loop. Crucially, these mechanisms are not designed to generalize the framework across all LLMs, but to preserve the integrity of simulation cycles while systematically exposing model limitations.

Fallback in Weekly Planning. At the beginning of each simulation cycle, the agent attempts to generate a complete weekly plan using an LLM conditioned on long-term memory and identity. If the model fails to return a valid structured response (e.g., due to invalid syntax or incomplete fields) after three attempts, a fallback plan is automatically synthesized:

$$\mathrm{If}\;{\mathrm{LLM}}_{\mathrm{weekly}}\left(I,M^{l}\right)\;\mathrm{fails}\;\geq 3\;\mathrm{times}\Rightarrow \mathrm{Plan}=\mathrm{RandomPlan}\left(I\right)$$

This plan is generated using pseudo-random parameters within the constraints of the agent’s identity (e.g., allowed transport modes and preferred departure windows). While not semantically aligned, it ensures continuity and prevents simulation interruption.

Fallback in Daily Planning. During short-horizon adaptation, if the daily plan cannot be revised successfully (e.g., due to LLM timeout, ill-formed JSON, or empty response), the agent retains the original plan for that day:

$$\mathrm{If}\;{\mathrm{LLM}}_{\mathrm{daily}}\left(I,M_{t}^{s},p_{t}^{\mathrm{init}},E_{t}\right)\;\mathrm{fails}\;\geq 3\;\mathrm{times}\Rightarrow p_{t}=p_{t}^{\mathrm{init}}$$

This fallback mechanism models real-world behavioral inertia, whereby individuals often adhere to their original intentions in the absence of compelling new information.

Model Evaluation and Design Implications. Fallback activations are logged and monitored as part of the model evaluation process. Preliminary internal testing with multiple LLMs revealed varied robustness profiles: models such as Qwen2.5-7B exhibited low fallback incidence, whereas others systematically failed to produce valid structured plans, leading to repeated planning breakdowns. These observations informed the architectural design of fallback mechanisms as diagnostic thresholds, rather than corrective tools. By embedding these strategies within the cognitive loop, the framework remains operational even when external reasoning fails, enabling both reliable long-term simulations and transparent evaluation of LLM suitability in agent-based mobility environments.

### 4.5. Illustrative Example

To illustrate the functioning of the proposed cognitive framework, we present the case of administrativeworkerm4, a 32-year-old office worker with a strict arrival deadline of 08:45 AM. This agent prioritizes punctuality and reliability over cost, comfort, or environmental impact.

Initial Planning and Exploration: In Week 1, the agent generated its first weekly plan based solely on its semantic identity, as no long-term memory existed. The plan included mostly taxi trips with slight variations in departure time and a single bus trip on Thursday. While the taxi journeys resulted in early arrivals, the bus trip led to lateness. This prompted a reflective evaluation of modal reliability under time constraints.

Adaptation and Strategy Consolidation: Informed by short-term memory and the abstracted insights from Week 1, the agent adopted a fully taxi-based strategy in Week 2, standardizing departure at 07:32 AM. This pattern persisted into Week 3 and Week 4, consistently yielding early arrivals and stable execution. A temporary shift to bus occurred during a disruption in Week 3, demonstrating limited but context-aware adaptation.

Reflection and Memory Abstraction: At the end of each week, the agent consolidated its experience into long-term memory. These abstractions guided future planning and reinforced successful routines. Across four weeks, the agent transitioned from exploratory behavior to a stable strategic pattern, while retaining the flexibility to fall back to alternative modes when disruptions occurred. Full daily outcomes and memory entries for this case are provided in Appendix B.

## 5. Experimental Setup and Simulation Design

This section presents the experimental setup used to validate the proposed cognitive architecture for urban mobility simulation. Our goal was to evaluate how LLM-based agents behave under both stable and disrupted transport conditions, using the Qwen2.5-7B model as the primary planner. This model was selected because it provides a strong balance between reasoning capacity and computational efficiency. In preliminary tests, it achieved a failure rate below 6% in generating structured, semantically consistent travel plans, while being substantially less resource-intensive than larger LLMs. This trade-off made it particularly suitable for multi-agent simulations at scale.

Each agent represented a transport user with semantic identity, episodic memory, and modal preferences. Over a 20-day simulated period, agents generated daily travel plans via LLM-based reasoning and interacted with transport providers instantiated in SimFleet.

Behavioral patterns, adaptation to disruptions, and alignment with user profiles were systematically assessed.

### 5.1. Time Scale and Simulation Acceleration

To enable practical execution times while maintaining behavioral realism, the simulation operates under an accelerated temporal scale. Specifically, a scale ratio of 4.3 is applied, meaning that one minute of simulated urban time corresponds to approximately 4.3 s of real-time computation. To ensure tractability of the simulation, a temporal acceleration ratio of 4.3 was applied, corresponding to 1 min of simulated time = 4.3 s of real time. This ratio was calibrated to balance computational efficiency and behavioral realism: it allows trip durations to remain within realistic temporal ranges while ensuring that large-scale multi-agent experiments can be executed without prohibitive computational costs. Consequently, a full simulation day spanning six hours (06:00 AM to 12:00 PM) requires around 26 min of real-world time to complete. All spatial and temporal parameters, including agent velocities and service frequencies (e.g., bus arrivals every 30 simulated minutes), are proportionally adjusted to preserve consistency within the compressed timeframe. For example, an agent experiencing a 42-s real-time taxi wait corresponds to a delay of approximately 10 min in simulated urban time. This acceleration approach ensures computational feasibility for large-scale agent populations while preserving interpretability and semantic plausibility in observed mobility behaviors.

### 5.2. Experiment Overview

A total of 320 cognitive agents were simulated in a synthetic environment modeled on Valencia, Spain, a medium-sized European city with multimodal transport infrastructure. The agents engaged in daily planning-execution-reflection cycles as defined in Section 4, generating structured JSON travel plans based on long-term identity, short-term memory, and environmental context. Two experimental scenarios were analyzed:

- A baseline scenario, where all services operated normally;
- A severe disruption scenario, in which 80% of taxis were deactivated for five consecutive days.

An intermediate condition involving 40% taxi availability was also simulated. However, the behavioral outcomes observed under this partial disruption closely mirrored those recorded during normal operations, exhibiting minimal divergence in modal choices or adaptation patterns. To maintain analytical clarity and highlight the contrasts between stable and critically disrupted environments, we focus the discussion exclusively on the full-service and severe disruption scenarios. In addition to these, we also performed an ablation study under the same disruption scenario to isolate the effects of each memory module. Three variants—A1 (no memory), B1 (short-term memory), and Full (short- and long-term memory)—were compared over 20 days (6400 trips); results are reported in Section 6.3.

### 5.3. Simulation Environment and Modalities

The urban simulation included three main transport modes:

- Bus: 10 vehicles operating along Line 10 of the public urban bus of Valencia;
- Taxi: 64 agents providing on-demand service with an average wait time of 14 min (simulated time) under baseline conditions;
- Walking: Always available and cost-free, with no infrastructure constraints.

**Figure 4:**
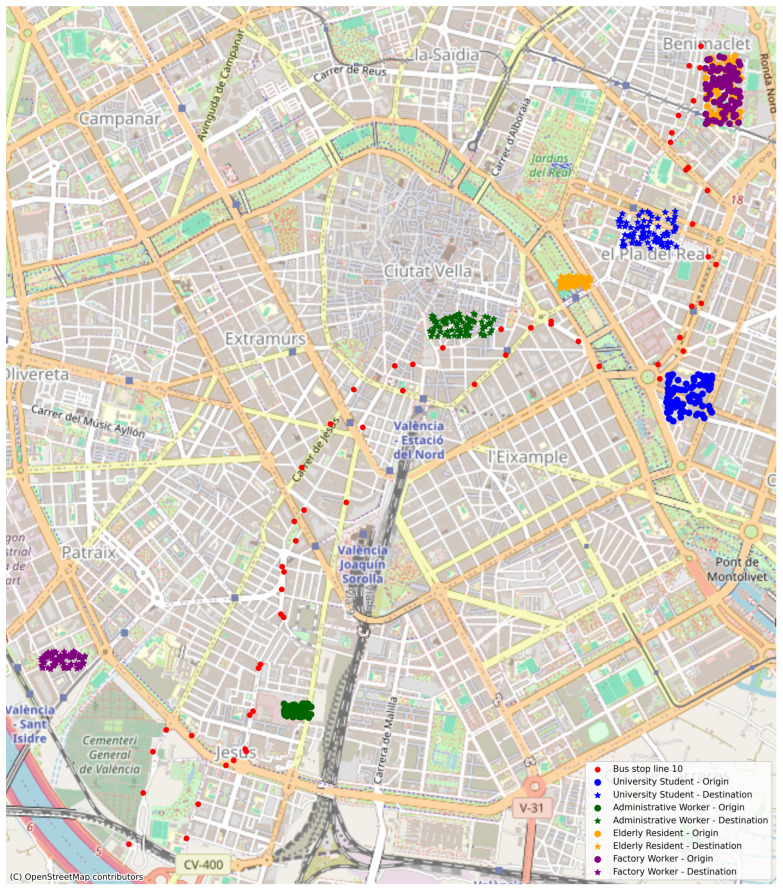
Spatial distribution of agent origins and destinations by user profile, overlaid on Line 10 bus stops. Red dots indicate bus stops; other markers denote profile-based origin and destination clusters.

Although SimFleet supports richer modal configurations (e.g., electric scooters, car-sharing), we deliberately constrained this study to a tri-modal setup to reduce complexity and isolate LLM-driven planning under uncertainty. Future work will extend this framework to incorporate additional mobility options. The spatial distribution of agents was designed to reflect realistic urban positioning by user type. Each profile was assigned a typical residential zone (origin) and a daily destination aligned with their occupational purpose. A small spatial jitter was applied for visual dispersion without altering travel distances. Figure 4 illustrates the resulting layout.

### 5.4. User Profiles and Behavioral Parameters

Each agent was instantiated with a semantic identity encoding sociodemographic attributes, modal preferences, income level, and activity schedules. The design of the four archetypes—University Student, Administrative Worker, Elderly Resident, and Factory Worker—is grounded in empirical mobility literature and reflects stylized behavioral traits extracted from recent survey-based studies. See specifically Table 4:

The full input data for the simulation is defined through structured configuration files, which specify fleet operators, transport modes, and user profiles with sociodemographic attributes and modal preferences. These files are version-controlled and openly available in a public repository (https://github.com/cvcalderon/simfleetdatabridge_config, accessed on 17 July 2025), ensuring transparency and reproducibility. Detailed examples of these configurations and their integration with the SimFleet simulator are provided in Appendix A.

- Students tend to exhibit high price sensitivity and environmentally conscious choices, often favoring walking and public transport [17].
- Administrative workers prioritize reliability and punctuality due to fixed office schedules [19].
- Elderly residents often value comfort and low walking effort, showing variable preferences depending on service availability [17,18].
- Factory workers, while typically budget-conscious, may prioritize time reliability when shift constraints are strict, especially under uncertain public transport performance [18].

The behavioral parameters were not drawn from a specific Valencia dataset but were constructed as stylized prototypes informed by recent survey-based mobility studies. In particular, priorities such as cost-sensitivity among students, punctuality requirements among administrative workers, comfort preferences among elderly residents, and the trade-off between time and cost for factory workers were parameterized from empirical findings reported in USA urban mobility surveys [17,18,19]. Travel distances were defined to reflect typical urban commute ranges (1–9 km) in medium-sized European cities, consistent with reports from Valencia’s metropolitan mobility plan. Income levels and modal expectations were aligned qualitatively with these archetypes rather than calibrated to statistical distributions. In this way, the profiles serve as empirically grounded behavioral testbeds, designed to examine whether cognitive agents reproduce differentiated and interpretable mobility behaviors under both stable and disrupted conditions.

### 5.5. Illustrative Example: University Student Agents

**Figure 5:**
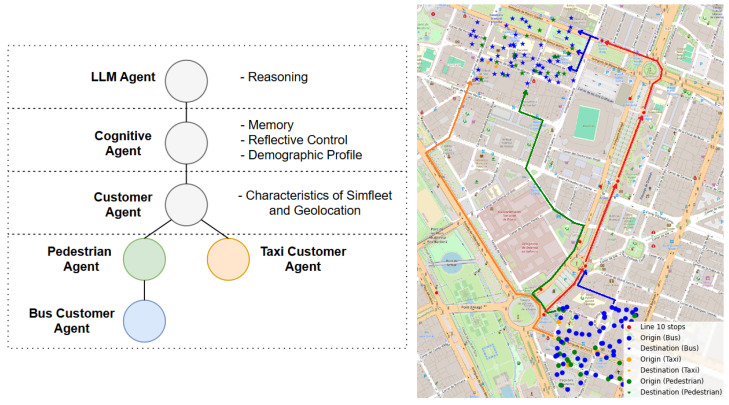
Illustration of heterogeneous cognitive agents and their spatial interactions for the University Student profile on simulation day 14. The figure illustrates how spatial environments condition feasible options, while cognitive reasoning drives the final modal choice.

To illustrate how abstract cognitive agents are instantiated in SimFleet and shaped by spatial constraints, Figure 5 presents the case of University Students. On the left, the hierarchical decomposition shows how the LLM-driven cognitive agent (reasoning, memory, reflection) is instantiated as a Customer Agent in SimFleet, operationalized through concrete roles such as pedestrian, bus passenger, or taxi user. This representation highlights that final behaviors emerge from the interplay of cognitive reasoning, semantic identity, and the operational rules embedded in SimFleet. On the right, the map shows the geolocated deployment of these roles within Valencia’s Line 10. Pedestrian and bus trips share almost identical origin–destination distributions, reflecting short commutes of 1–2 km. This overlap indicates that both modes are spatially interchangeable, and the eventual predominance of walking over bus use arises not from geography but from cognitive evaluation of cost and reliability. By contrast, taxi trips concentrate on fewer destinations, suggesting that taxis are mobilized primarily as punctuality-driven fallbacks when time reliability is at risk.

It is important to note that only the user-side agents (instantiated as SimFleet Customer Agents) embed the LLM-driven cognitive architecture. Service-side entities such as Bus, Taxi, and Bus Stop agents retain their standard SimFleet implementation, providing the structural and operational environment in which cognitive agents interact. This separation ensures that heterogeneity and adaptation arise from the reasoning processes of user agents, while transport services remain governed by consistent infrastructural rules.

Overall, the figure demonstrates how spatial interactions delimit the feasible choice set of each profile, while the cognitive architecture determines which option is ultimately adopted under stable or disrupted conditions.

These profiles were not intended as representative demographic clusters but as empirically informed behavioral prototypes designed to test whether semantic agent reasoning, memory, and adaptation mechanisms can produce differentiated and interpretable modal behaviors.

In addition, each agent generated an initial weekly plan and subsequently adapted daily plans using memory and context. Modal decisions were not predefined but emerged from LLM-driven reflection and situational feedback. This setup enables the investigation of emergent, profile-sensitive modal behaviors across stable and perturbed conditions, forming the basis for the experiments described in the following section.

## 6. Experimental Results

This section presents the empirical results of the 20-day simulation conducted with Qwen2.5-7B as the cognitive planning engine. We evaluate the capacity of the proposed framework to generate semantically coherent and context-aware travel behaviors under both stable and disrupted urban transport conditions. The analysis is divided into two main scenarios. The first examines baseline behavior when all transport services are fully operational. The second focuses on a severe disruption—an 80% taxi strike—introduced during the third week, which tests the agents’ ability to adapt to system-level perturbations. For each scenario, we study modal distribution across profiles, evolution over time, waiting times, and behavioral divergence, providing insight into the LLM’s decision-making capabilities and the role of memory-driven reflection.

### 6.1. Baseline Behavior: Profile-Driven Modal Evolution

**Figure 6:**
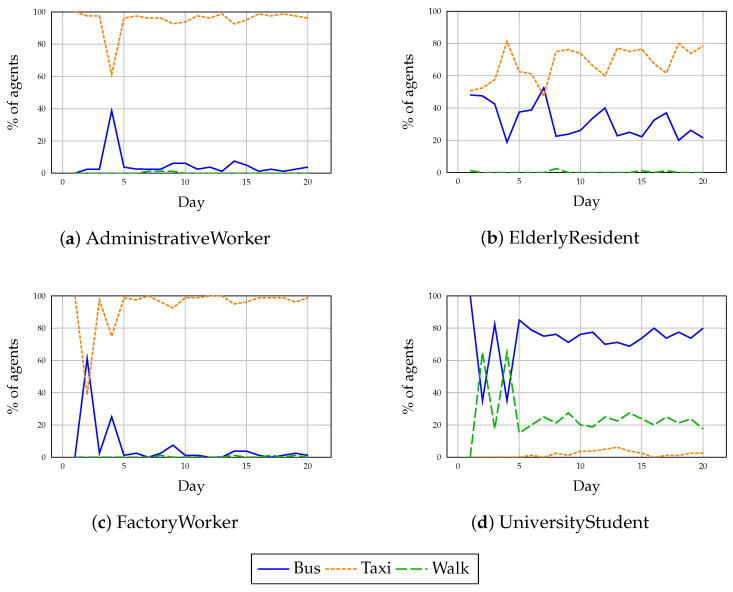
Evolution of transport mode selection under baseline conditions for each user profile (percentages).

Under normal operating conditions, agents exhibited the ability to converge towards semantically coherent travel behaviors that reflected their identity, mobility priorities, and environmental constraints. Figure 6 presents the evolution of transport mode usage over the 20-day simulation for each user profile. Despite initial exploration phases, agents generally stabilized their decisions by day 5, forming habitual patterns through the interaction between experience accumulation and memory-driven reflection mechanisms.

Administrative Workers predominantly selected taxis from the outset, maintaining a stable usage rate above 75% after a minor exploration event. Notably, on day 4, a transient surge in bus usage (31%) occurred, reflecting short-term experiential learning, but taxi preference was quickly re-established as memory consolidation favored time-efficient transport. This behavior aligns with the workers’ prioritization of time efficiency and reliability over cost considerations.

Elderly Residents displayed the highest modal flexibility among all profiles. Throughout the simulation, taxis remained the preferred mode (60–70%), but a substantial minority consistently utilized buses (18–42%). Minor daily fluctuations suggest that the reflection mechanism enabled dynamic balancing between comfort and moderate adaptability. Walking remained virtually absent (<2%), consistent with the comfort-driven nature of this demographic.

Factory Workers showed the most significant deviation from expected modal behavior. Contrary to budget-driven expectations, taxi usage dominated the early days (up to 80% on day 1), with a notable bus usage spike (49%) on day 2. However, convergence towards sustained bus usage was not achieved. This persistence of taxi preference, despite long distances (8.6 km) and low income, indicates that immediate time savings and favorable short-term outcomes influenced the LLM’s reflective reasoning more strongly than economic constraints.

University Students initially demonstrated an unexpected preference for bus transport, with 80% usage on day 1. This behavior, misaligned with their eco-budget profile and short travel distance (1.8 km), was rapidly corrected by day 2 following memory updates, with over 50% of agents switching to walking. From day 3 onwards, students maintained a mixed but semantically plausible modal pattern, favoring walking (12–22%) and bus (55–68%), with taxi usage remaining marginal (<5%). This progression reflects the system’s capacity for experience-informed adaptation.

Overall, these results validate that the cognitive framework enables emergent, profile-aware planning behaviors without the need for hardcoded rules. Agents demonstrated both habit formation and limited exploration, influenced by environmental feedback and memory-driven reflection processes. Minor deviations highlight the nuances of LLM-based decision-making, where trade-offs between multiple priorities can lead to non-intuitive but contextually defensible behaviors. This interpretation is consistent with the modal evolution curves in Figure 6, where short-lived deviations (e.g., the day-2 bus spike among Factory Workers or the initial over-reliance on buses by Students) gave way to stabilized, profile-aligned patterns within the first simulation week.

### 6.2. Disruptive Event: Taxi Strike and Adaptive Response

To evaluate the adaptability of the cognitive agents, a severe disruption was introduced during simulation days 11 to 15: an 80% reduction in taxi availability. Specifically, only 13 out of the original 64 taxi agents remained operational, substantially increasing wait times and reducing the reliability of on-demand transport services. All waiting times reported in this section are expressed in simulated minutes, following the accelerated time scale defined in Section 5. In SimFleet, the taxi strike was operationalized by deactivating 80% of taxi agents, leading to significantly longer delays in agent pickups. Agents perceived the disruption indirectly through extended waiting periods and travel outcome feedback, rather than via explicit event notifications. Consequently, any behavioral adaptation emerged solely from experiential learning and memory-based reflection, without predefined responses.

**Figure 7:**
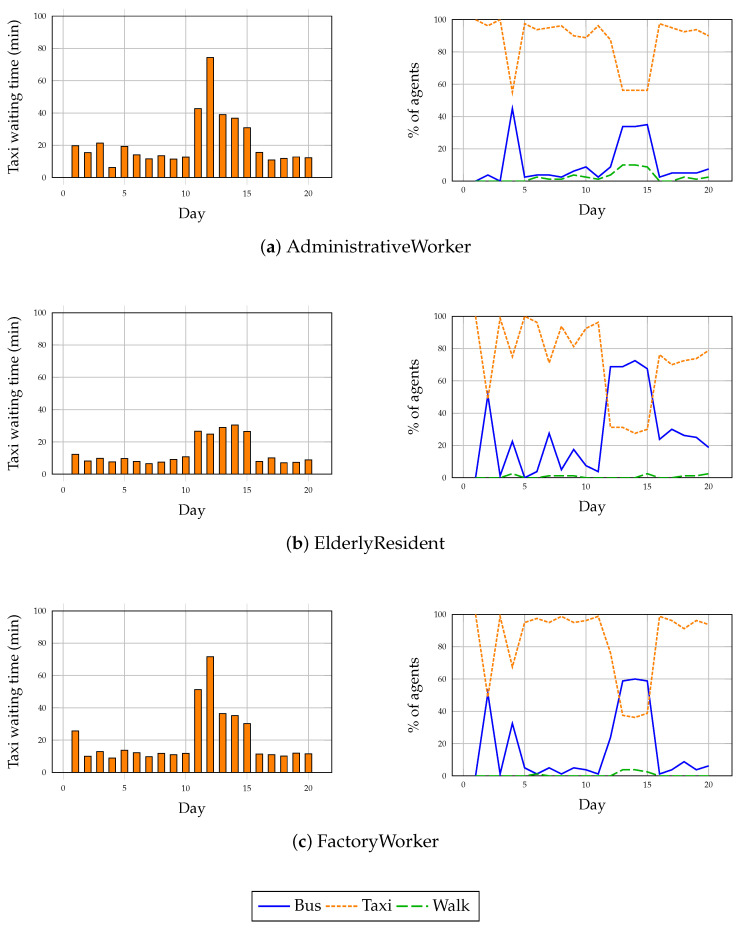
Transport behavior and taxi usage across different user profiles. Line charts represent mode selection over time; bar charts indicate average taxi waiting times per day (expressed in simulated minutes, as defined in Section 5).

Figure 7 presents the evolution of transport mode selection alongside the variation in taxi waiting times for each profile impacted by the strike. The results reveal differentiated adaptation behaviors across profiles:

Administrative Workers demonstrated the most effective adaptation. Following a sharp increase in taxi waiting times to approximately 78 simulated minutes by day 12, around 27–28% of these agents shifted from taxi to bus usage between days 13 and 14. This modal shift persisted throughout the disruption, reflecting successful memory-driven reflection and strategic re-planning. Once taxi services normalized after day 15, a partial reversion to taxi usage was observed, although bus usage remained slightly elevated compared with baseline conditions.

Elderly Residents exhibited moderate adaptation. A pre-existing tendency towards mixed mode usage allowed some resilience, with bus utilization increasing slightly (from 55% to nearly 58%) during the disruption window.

However, approximately 40–45% of elderly agents continued to attempt taxi use despite waiting times peaking above 30 simulated minutes, reflecting a strong comfort-based inertia that moderated their responsiveness to service degradation.

Factory Workers showed notable but temporary adaptation. Initially highly reliant on taxis, a marked shift to bus transport occurred from day 12 onwards, with bus usage increasing from 19% to approximately 47–48% by day 14. However, this adaptation proved unstable: immediately after service recovery, workers reverted almost entirely to their original taxi preference (79% taxi usage by day 16). This indicates that the disruption induced short-term behavioral changes but failed to produce lasting habit reconfiguration.

University Students, who predominantly relied on walking and bus transport modes even under baseline conditions, were minimally affected by the taxi strike and thus are not included in the detailed adaptation analysis.

Overall, while all affected profiles demonstrated some level of reactive adaptation during the disruption, the persistence and consolidation of alternative strategies varied significantly. This highlights the nuanced interplay between identity-driven priorities (e.g., comfort) and reactive experiential learning within the cognitive agent framework.

### 6.3. Ablation Study

Ablation studies are performed with the intention of evaluating the contribution of each individual component to the overall performance of a system. The proposed architecture consists of distinct components: Agent representation and identity, the LLM for decision-making, the Simulator, which executes generated plans, and, finally, two different memory components. In the context of the proposed architecture, the agent and simulator components cannot be decoupled, as they are the basis of the architecture. On the other hand, the deactivation of the LLM component would be unaligned with the scope of the paper. Thus, the ablation study is designed to evaluate the impact of the memory modules.

To discern the individual impact of each memory module, the experimentation presented in Section 6.2 is repeated using three framework variants. Thus, each variant has been evaluated across 20 days of simulation (320 agents, 6400 trips). The selection of transport modes—bus, taxi, and walking—remained consistent with those adopted in the previously detailed non-ablation experiments. The evaluation of these experiments will be performed based on the adequacy of the framework’s decision-making. In the context of the case study, this will be represented by the number of agent journeys that are successfully completed within the desired time range. The hypothesis motivating this ablation study is that each individual memory component contributes to improving agent decision-making, thus being rightfully added to the proposed architecture to reproduce dynamic and realistic agent behavior in simulations.

In the following, we list and describe the framework variations:

- A1 (no memory): The system architecture is adapted for its operation without the memory components. Agent representation lacks short and long-term memories. Agents base their decision-making on Long-Horizon Weekly Plans, generated at the beginning of each 5-day work week. The outputs of the simulation are not taken into consideration; that is, there is no daily reflection, no re-planning, and no pattern abstraction.
- B1 (short-term): The system architecture of A1 is extended with the inclusion of the short-term memory component. Agents begin applying their Long-Horizon Weekly plans, but, at the end of each simulated day, their simulation results are taken into account to apply Daily Reflection and Re-planning for the subsequent day, if necessary.
  However, no pattern abstraction is applied after each 5-day period. Thus, short-term memory is simply cleared out after this period, and no long-term learning is consolidated.
- Full: The system architecture of B1 is fully extended to include the long-term memory component. For the Full scenario, the architecture is the same one employed for the experiments of Section 6.1 and Section 6.2. Agents apply both reflection and re-planning after each simulated day and pattern consolidation after each 5-day period.

Table 5 summarizes punctuality outcomes for each variant under the disruption scenario (taxi strike). Results are presented aggregating the outcomes of all agents, regardless of its profile archetype, as the alignment of their behavior to their profile was outside the scope of the ablation study.

The ablation study demonstrates a clear, stepwise improvement in agent performance as cognitive components are added. Introducing short-term memory and daily reflection (B1) reduces late arrivals by 10.9 percentage points (from 34.25% to 23.35%) and decreases failed trips (“Incomplete”) by 3.0 points (from 4.3% to 1.3%). Further incorporation of long-term memory and weekly pattern abstraction (Full) lowers late arrivals to 14.75% and achieves an overall completion rate of 98.20%. These improvements stem primarily from the planner’s ability to refine critical decision variables—such as departure time and mode choice—based on recent outcomes, while memory mechanisms ensure that successful strategies are consolidated and reused over multiple cycles.

In the baseline (A1), agents re-execute a fixed plan and lack feedback: delays or failures on one day go uncorrected, leading to repeated tardiness and trip cancellations. In B1, episodic memory captures the previous day’s travel outcome, enabling daily re-planning that can shift the departure earlier or select a more reliable mode. Pattern abstraction in the Full model consolidates multi-day insights, biasing future weekly plans towards historically robust choices. Although the Full variant greatly reduced late arrivals, its trip completion rate was slightly lower than B1 (98.2% vs. 98.7%), as long-term habit formation can occasionally reinforce strategies that underperform during unexpected disruptions. This highlights the trade-off between robust habits and short-term adaptability in memory-driven architectures.

### 6.4. Interpretation and Design Insights for Memory-Driven Cognitive Agents

The results from both baseline and disruption scenarios provide critical insights into the effectiveness and limitations of the proposed cognitive framework for urban mobility simulation. First, the baseline experiments confirmed that agents could form stable, profile-aligned behavioral patterns without hardcoded modal rules. Modal choices emerged organically through reasoning processes based on identity attributes, environmental context, and experiential feedback. Small exploratory phases observed during early simulation days further reinforce the framework’s capacity for dynamic adaptation rather than deterministic behavior. Under disruptive conditions, all affected profiles exhibited some degree of reactive adaptation in response to environmental degradation, with adaptation emerging purely from experience-based perception of delays, without external signaling.

Administrative Workers demonstrated strong and sustained modal shifts towards public transport, while Elderly Residents showed moderate adaptation with taxis and buses. Factory Workers exhibited notable but short-lived reallocation behavior, and Students rapidly converged to walking and bus use after initial exploration. These differentiated responses illustrate that agent cognition is not uniformly optimal but realistically bounded by identity structures, short-term memory, and reflection quality. The emergence of both resilient and inertia-driven behaviors reflects a desirable heterogeneity in agent reactions, enhancing the realism of mobility simulations. This phenomenon is aligned with recent understandings of bounded rationality in cognitive systems [30]. Moreover, the agents’ partial and sometimes reversible adaptations highlight the limitations of memory-driven reasoning without explicit future prediction mechanisms. Although agents reflected on daily experiences, the absence of anticipatory mechanisms meant that their responses were primarily reactive and bounded by immediate feedback rather than forward-looking. This bounded rationality mirrors real-world urban mobility, where adaptations typically occur after disruptions and are conditioned by established routines.

Overall, the cognitive framework demonstrates strong potential for simulating complex human-like behaviors in urban mobility contexts. Empirically, the framework yielded stable routines under normal conditions and reactive flexibility under disruptions, consistent with bounded-rational behavior in urban mobility. However, the results also point to areas for future enhancement, particularly regarding anticipatory decision-making and the consolidation of new behaviors beyond short-term adaptation windows. These insights inform both the immediate conclusions and avenues for further research, as discussed in the next section.

## 7. Discussion

Based on the study and experiments conducted, the following discussion highlights key insights and challenges observed in the use of LLM-driven cognitive agents.

### 7.1. Limitations of LLM-Driven Cognitive Agents

While the cognitive architecture proposed in this work demonstrates strong capabilities in enabling experience-based behavioral adaptation, several limitations intrinsic to the use of LLMs must be critically acknowledged.

Robustness. Despite the integration of structured fallback mechanisms, the robustness of the cognitive agents remains partially dependent on the underlying LLM’s performance. Failures such as ill-formed outputs, incomplete planning structures, or semantic incoherence occasionally occurred, particularly when system resources were constrained or prompt ambiguity increased. Fallback strategies preserved continuity but occasionally introduced noise into the data.

Scalability. Each agent independently invokes the LLM for weekly planning, daily adaptations, reflections, and memory abstractions. While this architecture supports rich individualized cognition, it incurs substantial computational cost. Scaling the system to thousands of agents would significantly amplify demands on processing power, memory, and, in production environments, financial cost, especially when using commercial LLM APIs. Parallelization and batching strategies could mitigate some of these constraints, but core scalability remains a structural challenge.

Latency. Although simulations in this study operated asynchronously, allowing relaxed timing constraints, LLM response times varied considerably depending on model size and system load. Latencies ranged from a few seconds to over a minute per decision call during high-load scenarios. For real-time simulations or highly interactive environments, such variability could undermine responsiveness and realism.

Dependence on External Models. The architecture inherently relies on third-party LLMs, either via APIs or local deployments. This introduces external dependencies, potential security risks, and limited control over model updates or deprecations. In critical applications—such as transport policy evaluation or emergency planning simulations—such dependencies could be problematic.

Behavioral Biases. A further limitation arises from potential behavioral biases embedded in LLMs, such as implicit preferences for specific transport modes or stereotypical associations that are not empirically grounded. Although our framework relies on semantic profiles and memory-driven adaptation, these latent biases may still influence decision-making in subtle ways. Future work could address this issue through model fine-tuning with domain-specific mobility data, bias correction mechanisms, or constrained prompting strategies, ensuring that simulated behaviors more closely reflect real-world diversity.

These limitations underline the importance of continued architectural innovation, model selection refinement, and system optimization to ensure that LLM-driven cognitive agents remain viable, scalable, and reliable tools for complex urban mobility simulation.

### 7.2. Interpretability and Behavioral Transparency

An important challenge inherent to the proposed cognitive architecture lies in the interpretability of agent behaviors and the transparency of underlying decision processes. While agents successfully exhibit emergent, profile-aligned modal patterns, the mechanisms through which individual decisions are formed remain partially opaque.

Emergent Versus Explained Behavior. Modal choices were coherent, but the specific contribution of memory, environment, or identity to each decision remains opaque. This complicates the validation and auditing of simulation results, particularly in applications requiring high levels of explainability, such as public policy simulation.

Challenge of Causal Attribution. Behavioral shifts observed in response to environmental disruptions are plausible and reflect experiential learning. However, attributing causality—whether a shift resulted from short-term dissatisfaction, cumulative experiential thresholds, or latent identity-driven heuristics—is difficult without intrusive model probing. The distributed influence of memory, environment, and identity introduces complex, non-linear dynamics that resist straightforward causal explanations.

Memory Abstraction Complexity. The transformation of episodic experiences into semantic memory patterns, although essential for behavioral generalization, further obscures interpretability. Abstracted patterns may amalgamate multiple experiential signals, making it challenging to reverse-engineer specific decision precedents.

Implications for Model Validation. Limited interpretability constrains formal model validation. While emergent behavioral trends can be statistically analyzed, understanding misalignments or rare behaviors at the agent level remains challenging. This suggests a need for enhanced monitoring tools, introspection modules, or constrained prompting strategies to facilitate greater behavioral transparency.

Insights from Ablation Study. The ablation experiments under the 80% taxi strike scenario (see Section 6.3) reveal that introducing episodic memory and daily reflection reduces the number of late arrivals by nearly 50%, while also almost eliminating failed trips (see Table 5). Further refinement through long-term memory lowers tardiness to less than 15%, maintaining a completion rate above 98%. These results confirm the role of memory in improving robustness under disruption. These findings highlight the importance of memory mechanisms in enhancing robustness under severe disruptions.

Addressing these challenges will be crucial for extending the utility of cognitive agents in domains where trust, accountability, and decision auditing are critical.

### 7.3. Deployment Considerations

The proposed framework was designed not only to model agent-level reasoning, but also to support the simulation of transport policy interventions. Although this study focused on validating the cognitive planning architecture under baseline and disruption conditions, the framework is readily extendable to applied scenarios such as route redesign, congestion pricing, and priority schemes for vulnerable users. For instance, future simulations could evaluate how agents reconfigure their routines in response to modified bus routes or service frequencies, or how low-income profiles adapt to the introduction of dynamic congestion charges. Since agent behavior is driven by semantic preferences, memory, and environmental context, such interventions can be tested in terms of behavioral acceptability, modal reallocation, and distributional equity.

From a deployment perspective, the system is technically compatible with historical and real-time urban mobility datasets—such as GTFS feeds, ride-hailing logs, or sensor-based traffic data—via integration with the SimFleet simulation engine. This facilitates alignment with real-world infrastructure and service parameters. Nevertheless, full-scale operational deployment would require further development in areas such as user interface design, data pipeline integration, model calibration, and institutional adoption. These aspects constitute important avenues for future translational research, linking cognitively enriched simulation with evidence-based policy evaluation.

### 7.4. Experimental Scope

While the cognitive framework and simulation architecture were designed to test the feasibility of memory-guided behavioral modeling, the experimental configuration was intentionally constrained. A limited set of four agent profiles and a 20-day simulation window allowed for controlled observation of emergent planning, adaptation, and routine formation. Broader validation requires larger populations, richer profiles, and longer horizons (40–60 days) to evaluate stability, reversibility, and emergent dynamics.

In future work, we plan to conduct controlled experiments that isolate and compare the impact of short-term memory, long-term abstraction, and reflective planning. This includes ablation studies using agents without memory or with fixed plans, in order to quantify the behavioral adaptability specifically attributable to memory consolidation.

## 8. Conclusions and Future Work

We evaluated the proposed LLM-based cognitive architecture (Section 4). The results show profile-consistent behaviors under baseline conditions and reactive, experience-driven adaptations during severe disruption, without hardcoded modal rules.

The experimental evaluation consisted of simulating 320 agents with diverse sociodemographic profiles over a 20-day period, using the SimFleet platform. Each agent generated weekly travel plans according to its identity and past experience, and adapted its daily choices in response to changing transport conditions, such as an abrupt 80% reduction in taxi availability. The results confirm that LLM-driven agents can converge toward profile-consistent behaviors and adapt reactively under severe disruptions, without relying on hardcoded rules. Importantly, the ablation study demonstrated the critical role of short-term and long-term memory in enhancing punctuality and trip completion rates. Even within a limited 20-day horizon, emergent routines and reactive adaptations were observed, validating the framework’s cognitive plausibility. The ablation confirmed that short-term reflection drives immediate adaptation, while long-term memory strengthens punctuality. Even if the current 20-day simulation allowed for the emergence of modal habits and reactive adaptation, we acknowledge that this time frame may not fully capture long-term behavioral consolidation or inter-week routine evolution. Extending the simulation period to 40 or 60 days will enable a more comprehensive study of stable pattern formation, disruption-induced behavioral shifts, and the resilience of learned strategies over time. Such extensions will also allow the framework to test memory degradation, behavioral reversibility, and slow habit reinforcement in more realistic cognitive timelines.

The architecture can also be applied to service providers such as fleet managers or bus operators. Service providers such as taxi fleet managers, bus operators, or infrastructure coordinators could equally adopt memory-driven planning and adaptation strategies. By integrating multi-horizon reasoning and experiential reflection, service agents could dynamically optimize operations in response to fluctuating demand, disruptions, or environmental changes, enhancing both system efficiency and resilience. Future work includes anticipatory reasoning, introspective explanations to improve transparency, and extending profiles to marginalized populations for equity-focused policy evaluation.

Scalability remains a critical focus: optimizing LLM invocation strategies, exploring lightweight model alternatives, and developing efficient batching mechanisms will be essential for simulating large-scale urban populations. Finally, extending the framework to simulate policy interventions, multimodal system designs, and coordinated agent behaviors across heterogeneous roles represents an exciting avenue for future research, bridging urban mobility simulation with cognitive modeling and artificial social systems.

## Figures and Tables

**Table 1:** Comparison of Agent-Based Modeling Paradigms for Mobility Simulation.

Feature	Rule-Based ABM	Hybrid ABM + ML	LLM-Based Cognitive Agent
Behavior rules	Static, expert-defined	Static + ML output	Dynamic, goal-driven reasoning
Adaptability	No	Partial (data-driven)	Yes (reflexive + contextual)
Planning horizon	None or single-step	Short-term prediction	Multi-horizon (week/day)
Memory use	None	No memory	Structured (episodic + semantic)
Disruption response	Manual rules	Implicit from data	Reflective re-planning
Learning over time	No	No	Yes (memory consolidation)
Demographic profiling	Limited	Optional features	Socio-demo profiles
Explainability	Rule-based traceable	Medium (white-box)	High (language-level)

**Table 2:** Comparison of Cognitive Capabilities in LLM and ABM Architectures.

Architecture	Memory	Planning	Adaptation	Profile Modeling
ABM [4]	No	No	No	No
ML-RF [7] /GBDT [21]	No	No	No	No
PateGAIL [6]	No	No	No	No
ExpeL [12]	Yes	Yes	Yes	No
MindMemory [27]	Yes	No	Yes	Partial
NeoPlanner [26]	Yes	Yes	Yes	No
Mobility-LLM [13]	Yes	No	No	Partial
MobGLM [8]	No	No	No	Yes
HMP-LLM [11]	No	Yes	No	No
Hou et al. [25]	Yes	No	Yes	No
Hatalis et al. [24]	Yes	No	Partial	No
TransCompressor [14]	No	No	No	No
Ours (This Work)	Yes	Yes	Yes	Yes

**Table 3:** Application Context in Mobility and Simulation Environments.

Architecture	Simulation Based	Disruption Handling	Mobility Domain
ABM [4]	Yes	No	Yes
ML-RF [7] /GBDT [21]	Yes	No	Yes
PateGAIL [6]	No	No	Partial
ExpeL [12]	No	Partial	No
MindMemory [27]	No	No	No
NeoPlanner [26]	No	No	No
Mobility-LLM [13]	No	No	Yes
MobGLM [8]	No	No	Yes
HMP-LLM [11]	No	Yes	Yes
Hou et al. [25]	No	No	No
Hatalis et al. [24]	No	No	No
TransCompressor [14]	No	No	Partial
Ours (This Work)	Yes	Yes	Yes

**Table 4:** Behavioral parameters for agent profiles, including typical one-way travel distance between home and primary destination.

N°	Profile	Modal Priorities	Distance	Expectation
80	University Student	Budget, Eco-friendly	1.8 km	Walk, Bus
80	Administrative Worker	Time, Reliability	4.0 km	Taxi, Bus
80	Elderly Resident	Comfort, Reliability	2.8 km	Bus, Taxi
80	Factory Worker	Time, Budget	8.6 km	Bus, Taxi

**Table 5:** Ablation metrics under 80% taxi strike (6400 trips). Metrics: Early = percentage of early arrivals; Late = percentage of late arrivals; On_time = percentage of on-time arrivals; Incomplete = percentage of trips not completed; Completion = percentage of completed trips.

Variant	Early	Late	On_Time	Incomplete	Completion
A1	58.95%	34.25%	2.50%	4.30%	95.70%
B1	71.95%	23.35%	3.60%	1.30%	98.70%
Full	81.05%	14.75%	2.40%	1.80%	98.20%

## Data Availability

No new data were created or analyzed in this study. Data sharing is not applicable to this article.

## Appendix A. Implementation and Execution Details

This appendix outlines the technical underpinnings for configuring, executing, and reproducing experiments using the SimFleet platform alongside the cognitive framework. It highlights the principal configuration structures and integration principles that underpin scientific transparency and reproducibility.

### Appendix A.1. SimFleet Configuration File

SimFleet scenarios are defined via structured JSON configuration files, with the central file being simfleet_config.json. This file specifies all essential simulation entities and their interrelations, including the following:

- fleets: Describes each fleet operator, specifying identifiers and fleet type (e.g., buses).
- transports: Lists individual vehicles or agents, detailing their type, operational class, strategy, location, speed, and other operational parameters.
- customers: Enumerates customer agents, each with attributes such as class, strategy, position, and destination.

This modular structure allows for flexible and precise definition of multimodal urban mobility scenarios, enabling scenario-specific adaptations and extensions.

### Appendix A.2. Framework Configuration File Structure

The cognitive framework employs additional JSON files to define simulation parameters and agent behaviors:

- framework_config.json: Specifies simulation environment settings, agent actions, AI model parameters (including LLM model selection and endpoints), and possible environmental events (e.g., special disruptions).
- profiles.json: Contains detailed agent profiles, including sociodemographic data, individual mobility preferences, and available transport modes, thus supporting heterogeneous simulation populations.

Collectively, these configuration files enable reproducible, modular, and extensible scenario definitions within the cognitive framework. Comprehensive documentation and examples are available in the configuration repository.

### Appendix A.3. Reproducibility and Integration

All scenario specifications within the cognitive framework are externalized in version-controlled configuration files, thereby facilitating full reproducibility of experiments. This design also enables seamless integration with external modules, such as alternative LLM providers or custom agent strategies, by permitting dynamic references to models, actions, and environments within the configuration files. This approach ensures that simulation setups, agent behaviors, and experimental conditions remain transparent and readily shareable.

The complete source code and sample configurations are available in the following repositories:

- https://github.com/javipalanca/simfleet (accessed on 17 July 2025)
- https://github.com/cvcalderon/simfleetdatabridge (accessed on 17 July 2025)
- https://github.com/cvcalderon/simfleetdatabridge_config (accessed on 17 July 2025)

## Appendix B. Memory-Guided Planning Case Study

This appendix presents detailed simulation outcomes for the agent administrativeworkerm4, covering all planning, execution, and reflection cycles over a four-week simulation. The case illustrates the memory-guided cognitive loop in full operation.

### Appendix B.1. Week 1: Exploratory Planning in Absence of Experience

As no long-term memory existed at the start of the simulation, the agent generated its weekly plan based exclusively on semantic identity. The plan showed exploratory features: taxis on most days, but one bus trip on Thursday, and small variations in departure time.

**Table A1:** Week 1 decisions and outcomes for administrativeworkerm4.

Day	Mode	Departure	Arrival	Reflection Summary
Monday	Taxi	07:32 AM	08:10 AM	Arrived 35 min early; reliable.
Tuesday	Taxi	07:32 AM	07:57 AM	Efficient; confirmed plan effectiveness.
Wednesday	Taxi	07:46 AM	08:09 AM	Consistent performance.
Thursday	Bus	08:21 AM	08:56 AM	Arrived 11 min late; questioned bus reliability.
Friday	Taxi	07:35 AM	07:58 AM	Early arrival; taxi strategy reaffirmed.

### Appendix B.2. Week 2: Taxi-Based Routine and Strategy Consolidation

Following the reflections stored in short-term memory and the abstracted long-term memory from Week 1, the agent shifted to a consistent strategy using taxis only, with standardized departure at 07:32 AM. All arrivals occurred between 07:57 and 08:09 AM, indicating stability.

Memory abstraction: “The user arrived early every day using taxis. This pattern meets punctuality goals and offers reliable travel duration.”

### Appendix B.3. Week 3: Disruption and Mode Shift

In Week 3, the simulated environment experienced a taxi shortage due to a disruption. The agent maintained its preferred plan initially, but on Thursday switched to a bus to avoid extensive delays. Reflections recognized the vulnerability of taxi reliance under disruption.

Memory abstraction: “Taxis are generally reliable but failed during disruption. A bus fallback may be warranted in high-risk periods.”

### Appendix B.4. Week 4: Recovery and Reconfirmation of Routine

In Week 4, the agent returned to its taxi-based strategy. Although one entry was marked as a bus trip, the reflection indicated that a taxi was actually used, reaffirming the pattern established in previous weeks. Arrivals remained consistently early (between 07:57 and 08:05 AM).

Memory abstraction: “The established routine continues to meet time constraints. Confidence in plan effectiveness is high.”

### Appendix B.5. Multi-Week Memory-Guided Adaptation

The weekly memory abstractions stored in long-term memory are as follows:

- Week 1: Taxis ensured punctuality; bus caused lateness.
- Week 2: Fully taxi-based strategy confirmed as reliable.
- Week 3: Taxi failure during disruption justified bus fallback.
- Week 4: Taxi routine reaffirmed; continued early arrivals.

### Appendix B.6. Behavioral Summary

The full cognitive planning loop was observed:

- Initial exploration without memory guidance.
- Formation of a stable modal strategy aligned with user identity.
- Experience-based adaptation during external disruption.
- Reinforcement and reuse of successful strategies via memory abstraction.

This case provides empirical grounding for the architecture’s mechanisms of reflection, adaptation, and memory-driven behavioral modeling.
